# Type 2 immunity‐driven diseases: Towards a multidisciplinary approach

**DOI:** 10.1111/cea.14029

**Published:** 2021-10-15

**Authors:** Dorian Hassoun, Olivier Malard, Sébastien Barbarot, Antoine Magnan, Luc Colas

**Affiliations:** ^1^ CHU Nantes, CNRS INSERM, l’institut du Thorax Université de Nantes Nantes France; ^2^ Department of Otorhinolaryngology and Head and Neck Surgery Nantes University Hospital Nantes France; ^3^ Department of Dermatology CHU Nantes, UMR 1280 PhAN, INRA Nantes Université Nantes France; ^4^ INRAe UMR_S 0892, Hôpital Foch Université de Versailles Saint‐Quentin Paris Saclay France; ^5^ Plateforme Transversale d'Allergologie et d'Immunologie Clinique Institut du Thorax CHU de Nantes Nantes France; ^6^ INSERM, CHU Nantes Centre de Recherche en Transplantation et Immunologie UMR1064 Nantes Université ITUN Nantes France

## Abstract

Asthma, atopic dermatitis and chronic rhinoconjunctivitis are highly heterogeneous. However, epidemiologic associations exist between phenotypic groups of patients. Atopic march is one such association but is not the only common point. Indeed, beyond such phenotypes, hallmarks of type 2 immunity have been found in these diseases involving immune dysregulation as well as environmental triggers and epithelial dysfunction. From the canonical Th2 cytokines (IL‐4, IL‐5, IL‐13), new cellular and molecular actors arise, from the epithelium's alarmins to new innate immune cells. Their interactions are now better understood across the different environmental barriers, and slight differences appeared. In parallel, the development of type 2‐targeting biotherapies not only raised hope to treat those diseases but also raised new questions regarding their true pathophysiological involvement. Here, we review the place of type 2 immunity in the different phenotypes of asthma, chronic rhinitis, chronic rhinosinusitis and atopic dermatitis, highlighting nuances between them. New hypotheses rising from the use of biotherapies will be discussed along with the uncertainties and unmet needs of this field.

## Graphical abstract

Heterogenic diseases as asthma, atopic dermatitis and chronic rhinosinusitis with nasal polyposis can be divided into multiple phenotypes. Such clinical presentations share common pathophysiological paths among with type 2 immunity play an important role. Better understanding of the mechanisms underlying type 2 responses allowed the successful development of specific therapeutic strategies. Indeed, biotherapies are now available to target key actors like the immunoglobulin E, the interleukin (IL) 5, the IL‐4 and IL‐13 pathway and more recently, the thymic stromal lymphopoietin. Even though those innovations improve the healthcare of type‐2 mediated diseases, unmet needs remain to be resolved. Graphic abstract created with BioRender.com

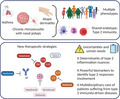

**Key Messages**

- Specific phenotypes of cutaneous and respiratory diseases share a common endotype: type 2 inflammation.
- Better understanding of type 2 inflammation leads to common innovative therapeutic strategies among such diseases.
- Uncertainties remain and critical unmet needs should be resolved to improve healthcare of such diseases.

## 1 INTRODUCTION

Atopic dermatitis (AD), allergic rhinitis (AR), chronic rhinosinusitis with (CRSwNP) or without (CRSsNP) nasal polyps and asthma share a complex interplay between a genetic background, a polarized immune response and the environment. Their increasing prevalence in urban areas in comparison with rural areas further highlights the role of environmental factors in their development.^1^ Interestingly, they can succeed themselves or coexist at the same time in a single individual throughout life. For example, atopic march describes the successive development of AD, food allergy, AR and allergic asthma during childhood.^2^ Nevertheless, such an association is not absolute, and if common pathophysiological mechanisms are shared, they are insufficient to explain the whole pattern's heterogeneity.

To address such heterogeneity, phenotypes, defined by the association of specific observable characteristics, were identified. Cluster analyses of large cohorts of patients demonstrated that clinical phenotypes could be isolated with different clinical presentations, clinical courses and responses to treatment.^3^ From those considerations, the concept arises that, behind such variable phenotypes, different physiopathological pathways could be deciphered. Such specific pathways, called endotype, were developed from omics data analysed by innovative bioinformatic tools.^4^

Type 2 inflammation is a particular endotype that plays an important dual role in environment‐related responses.^5^ On the one hand, it is involved in physiological responses against venom and helminths and in tissue repair.^6^ On the other hand, allergic and/or atopic diseases are prototypical examples of sustained and uncontrolled type 2 immunity.^7^ Data from studies of allergic diseases and animal models highlighted the critical roles of key cytokines, namely, IL‐4, IL‐5 and IL‐13, the production of specific IgEs and key cellular actors, principally, eosinophils and mast cells.^8^ In parallel with the endotype concept, the treatable mechanisms theory gave rise to specific biologics (monoclonal antibodies and small molecules) targeting such key pathways.

Here, we will discuss how type 2 inflammation is differentially implicated in several respiratory and cutaneous diseases. Next, an update of the new therapeutic strategies available will be presented along with the new hypotheses that come from its use in practice. Finally, we will highlight the uncertainties of this field and the unmet needs.

## 2 TYPE 2 INFLAMMATION: WHERE DOES IT FIT?

### 2.1 From phenotypes to endotypes

AD, AR, CRSwNP or CRSsNP and asthma have a broad definition, which highlights their heterogeneity. AR requires the demonstration of a causal link between atopy, allergen sensitization and allergen‐driven nasal symptoms. Conversely, CRS, a frequent upper airway condition (11% of the adult population), is defined by evidence of rhinosinusitis on computed tomography (CT) and/or by nasofibroscopy along with chronic nasal symptoms (≥12 weeks).^9^, ^10^ The same observation can be made with asthma, which is determined by respiratory symptoms related to variable airflow limitation in a variable bronchial inflammation setting.^11^ AD is clinically defined by chronic or relapsing eczematous skin lesions associated with pruritus.^12^ To reduce heterogeneity and help decipher the critical physiopathological pathways involved, clinical studies of such diseases have been performed to identify homogeneous phenotypic groups (Table 1).

**TABLE 1:** Main phenotypes of environment‐driven diseases implicating type 2 immunity

Barrier	Disease	Phenotype	Clinical characteristics	Biological characteristics	Associated comorbidities
Cutaneous	AD	***Extrinsic AD***	Early onset Atopic background Chronic relapsing course	High total and allergen‐specific IgE High rate of filaggrin mutations	**AR** **Allergic asthma** Food allergy
		***Intrinsic AD***	No atopic background	Normal total IgE levels and no specific IgE	NA
Respiratory	Asthma	***Allergic asthma***	Early onset Atopic background	High total and allergen‐specific IgE	**AR** **AD**
		***Nonallergic eosinophilic asthma***	Late onset Severe asthma	High blood eosinophilia High sputum eosinophilia	**CRSwNP**
		*Obesity‐related asthma*	Adult onset Obesity	Variable inflammatory components	Dysmetabolic syndrome
		*Noneosinophilic asthma*	Late onset	Paucigranulocytic or neutrophilic infiltrates	NA
	Rhinitis	***AR***	Early onset Atopic background	High total and allergen‐specific IgE	**AD** Allergic conjunctivitis
	CRS	***CRSwNP***	Adult onset High recurrence post polypectomy	High blood eosinophilia High mucosal eosinophilia IgE formation against *S*. *aureus* enterotoxins	**Severe eosinophilic asthma**
		*CRSsNP*	Early adult onset Less severe sinus lesions	Low eosinophilia Neutrophilic infiltration Overexpression of TGFβ	NA

Abbreviations: AD, atopic dermatitis; AR, allergic rhinitis; CRSsNP, chronic rhinosinusitis without nasal polyposis; CRSwNP, chronic rhinosinusitis with nasal polyposis.

The bold text indicates the most common phenotypes of disease and their most common associated comorbidities.

Several clinical characteristics to be considered, including age of onset, are crucial. Indeed, infancy onset of AD is strongly associated with the early onset of AR and asthma.^13^ In adulthood, a comparable association between AD and asthma can be found.^14^ Atopy, another critical phenotypic trait, is defined by the propensity of an individual to develop a specific IgE response against harmless antigens. As a part of AR definition, nasal symptoms need to be consistent with specific IgE production and/or cutaneous prick tests. Conversely, atopy and evidence of IgE‐related cutaneous symptoms are necessary to define extrinsic AD in exclusion of intrinsic AD. It is then mainly associated with other atopic diseases such as AD and allergic asthma.

Ethnic background has a differential impact on the expression of those diseases. For example, in AD, the clinical manifestation can be completely different among Asian, African American and European patients.^15^ In CRS, a multi‐centre study in Europe, Asia and Oceania demonstrated that lower predominance of eosinophils in nasal polyps and mixed cytokines pattern (Th1/Th2/TH17) could be found in Chinese population.^16^ In contrast, in asthma and AR such consideration does not seem to be critical. In parallel, some phenotypic characteristics are specific to the disease. In CRS, two phenotypes are described following nasal endoscopy: one with (CRSwNP) and one without (CRSsNP) nasal polyps with a ratio of approximately 1 to 4.^17^ In asthma, obesity is frequently associated with a specific severe presentation responding to personalized care (loss of weight, bariatric surgery when necessary, etc.).^18^

**FIGURE 1:**
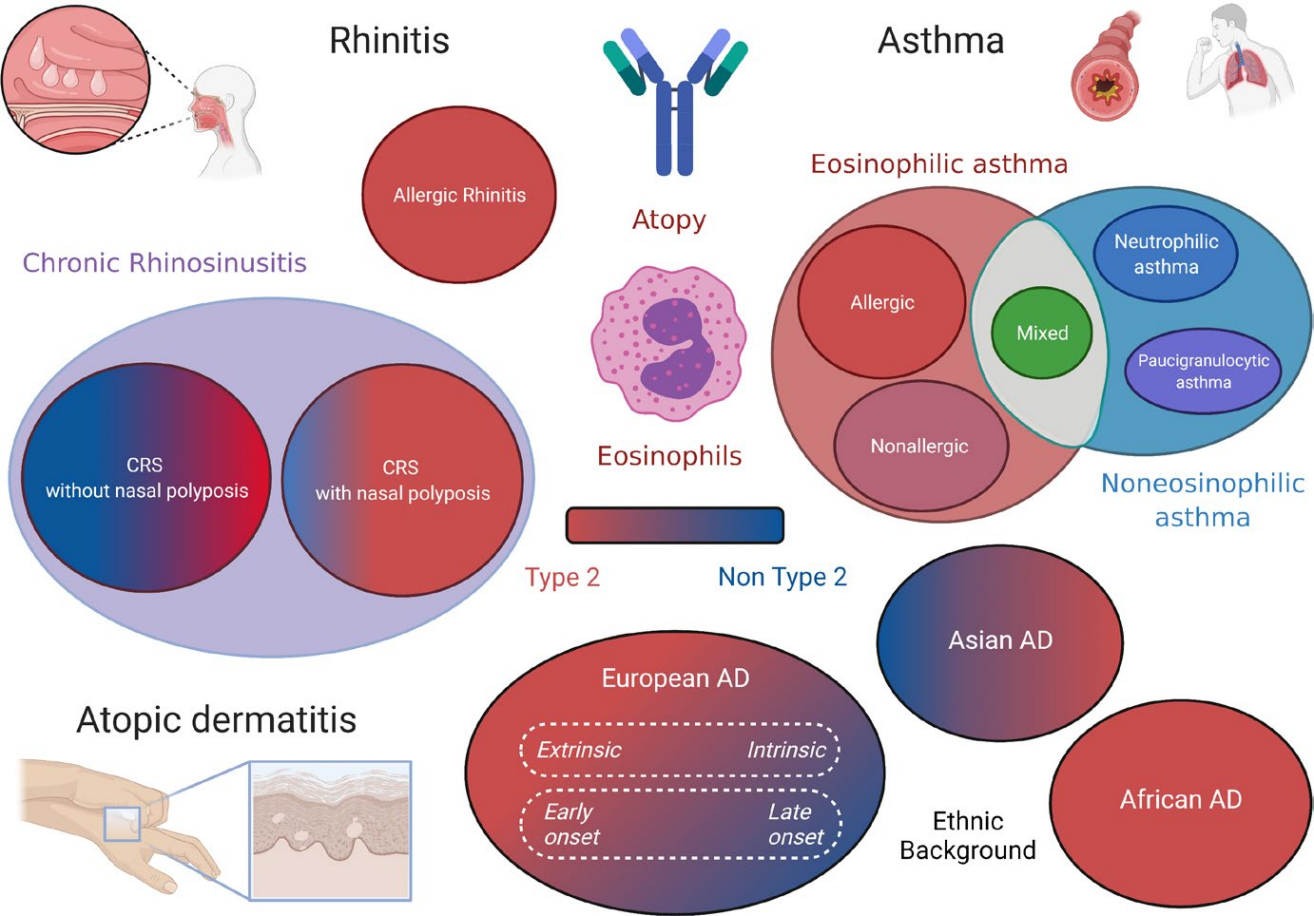
Phenotypic traits and main endotypes of environment‐driven diseases. Type 2 immunity is implicated only in a small proportion of patients affected by chronic rhinitis, asthma and atopic dermatitis. Created with BioRender.com

Beyond such phenotypes, endotypes have been discovered over the years thanks to cluster analyses and physiopathological assessments (Figure 1).

It has been highlighted that AD can be divided into several physiopathological pathways according to serum measures.^19^ Two high type 2 cytokine family clusters could be described depending on high levels of thymic stromal lymphopoietin (TSLP) or pulmonary and activation‐regulated chemokine (PARC). Interestingly, such clusters were differently associated with AD severity, the latter being associated with more severe disease.

In parallel, cluster analysis performed on a European cohort of patients suffering from chronic rhinosinusitis demonstrated that 2 main endotypes could be distinguished: an IL‐5‐high (eosinophilic) endotype linked to type 2 immunity and an IL‐5‐low (noneosinophilic)‐driven disease.^20^, ^21^ Eosinophilic chronic rhinosinusitis (ECRS) was then defined by an eosinophil count in nasal mucosa greater than or equal to 70 eosinophils/HPF (magnification, ×400). A strong association between CRSwNP and eosinophilic signatures has been described, consistent with that in other studies.^22^ Nasal polyps also have higher total and specific local IgEs associated with eosinophilic inflammation.^23^ Up‐regulation of the coagulation cascade and down‐regulation of fibrinolysis strongly induce abnormal fibrin deposition in nasal mucosa, and type 2 inflammation plays a central role in the imbalance of coagulation and fibrinolysis, further highlighting its role in polyp formation.^21^

In asthma, the natural course of the disease mainly depends on the involvement or not of an eosinophilic inflammation.^3^, ^24^ Eosinophilic asthma is defined by significant bronchial infiltration by eosinophils evaluated by sputum induction (>3% of cells). Blood hypereosinophilia (>500 mm^−3^) can be observed in approximately 10% of the overall population of asthmatic patients, and significant sputum eosinophilia can be observed in approximately 50% of the severe asthma population.^25^, ^26^ Allergic asthma and nonallergic asthma associated with CRS are examples of eosinophilic asthma.^27^, ^28^, ^29^ In those subpopulations, eosinophils are closely linked to exacerbation rate, control of the disease and quality of life. Noneosinophilic asthma includes neutrophilic asthma and pauci‐granulocytic asthma, which are less understood.^30^, ^31^

Some pathological paths that seem specific could be important in several fields. Filaggrin gene mutations (R501X and 2282del4) are closely linked to AD susceptibility, particularly in those with elevated serum total IgE.^32^ Interestingly, such mutations are also linked with asthma predisposition and severity.^33^, ^34^ Similar observations could be made between filaggrin gene mutations and sensitization and AR in meta‐analyses.^35^ Another example is the link between the microbiome and disease course. *Staphylococcus aureus* colonization and their specific IgE are associated with CRSwNP pathophysiology.^36^ A comparable observation can be made in AD lesion type‐ and severity‐linked *S*. *aureus* colonization.^37^, ^38^ It is also correlated with high IgE production and type 2 responses in AD.^39^, ^40^

Even though respiratory and cutaneous diseases seem to be completely different, epidemiologic associations of specific phenotypes can be found, and type 2 immunity hallmarks can be found.

### 2.2 Type 2 inflammation: Similarities and differences among diseases.

**FIGURE 2:**
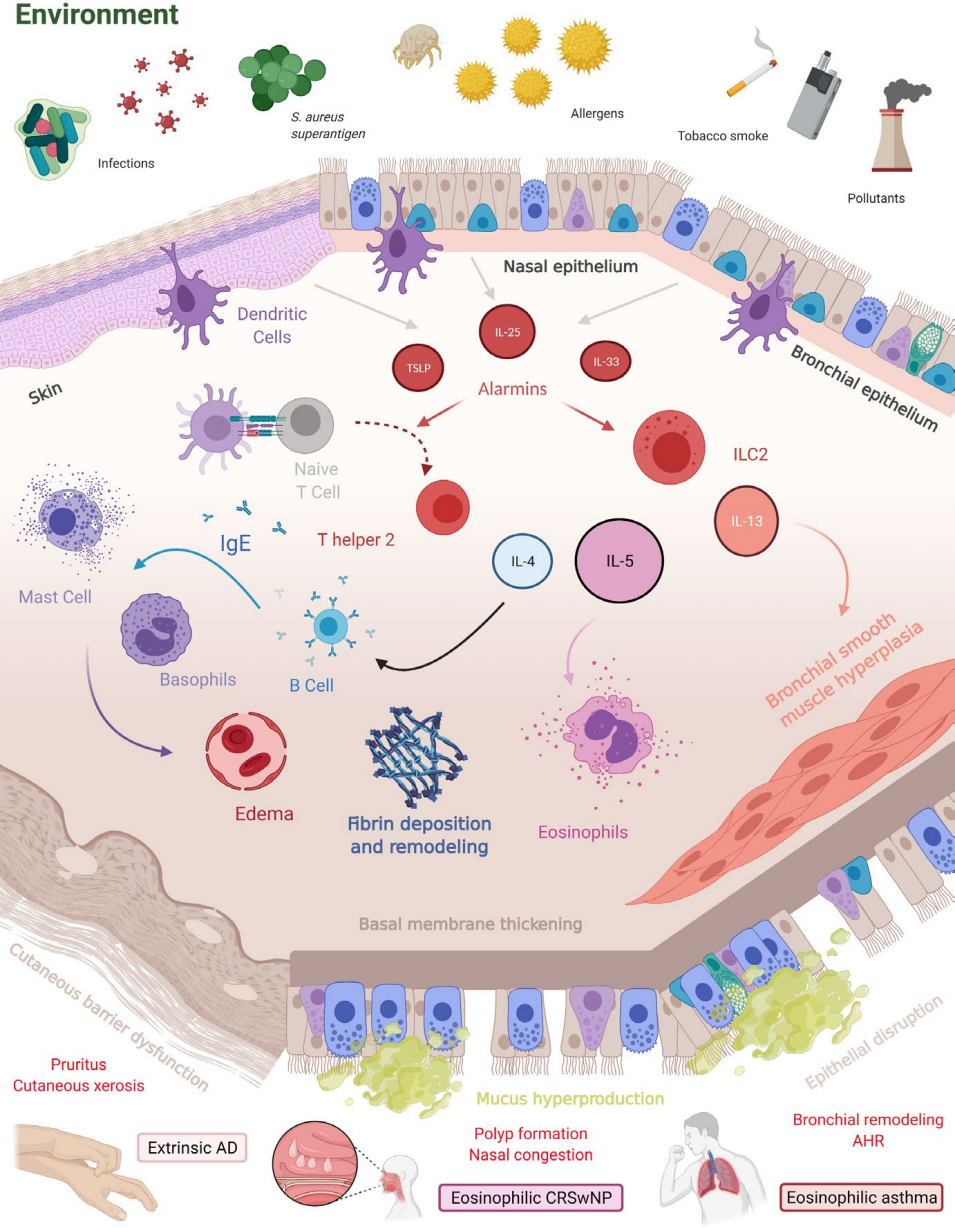
Type 2 inflammation in environment‐driven diseases. Diverse environmental aggression activates innate and adaptative immunity towards type 2 polarization. The epithelium plays an important role not only as an activator but also as a collateral target of inflammation. Eosinophilic infiltration and basophil and mast cell degranulation upon renewed stimulation induce oedema and critical tissue remodelling. Overall, tissue inflammation and epithelial dysfunction lead to hallmarks of cutaneous and respiratory symptoms. Abbreviations: AD, atopic dermatitis; AHR, airway hyperresponsiveness; CRSwNP, chronic rhinosinusitis with nasal polyposis; IgE, Immunoglobulin E, IL, interleukin; ILC2, group 2 innate lymphoid cell; TSLP, hymic stromal lymphopoietin. Created with BioRender.com

Though type 2 inflammation can be found in several diseases, complex interactions between the environment and cellular actors drive the observed nuances depending on the barrier (Figure 2).

Epithelial dysfunction is shared across type 2‐driven diseases. In AD, it is a multifactorial core characteristic including genetic factors such as filaggrin mutations.^41^ A dysfunctional cutaneous barrier leads to overstimulated damaging immune responses that weaken the epithelium, creating a vicious cycle.^42^ Recently, it has been demonstrated that IL‐13 alters the tight junctions of cutaneous and bronchial epithelia in AD and asthma, respectively.^43^, ^44^ Mature polyps from CRSwNP patients, defined by end‐stage remodelled polyps (stromal oedema, fibrin deposition, loss of epithelial cells), also present a down‐regulation of adhesion molecules in comparison with healthy mucosa.^45^ However, the epithelium is not only a passive physical barrier but also a potent modulator of immune responses through the secretion of alarmins.

Thymic stromal lymphopoietin from the IL‐7 family is able to activate Langerhans cells and skew them to a pro‐Th2 phenotype in AD.^46^, ^47^ Conversely, stimulation of the TSLP receptor increases IL‐4 secretion by CD4^+^ T cells from AD.^48^ TSLP from nasal polyps induces higher IL‐5 secretion by mast cells ex vivo, highlighting its important role in the upper airways.^49^ A similar consideration was made with higher TSLP epithelial expression found in bronchial biopsies from asthmatic patients in comparison with healthy volunteers.^50^ In parallel, autocrine type 2 processes can be found: stimulation by type 2 cytokines and TLR3 increases the ability of cultured human bronchial epithelial cells to be stimulated by TSLP.^51^

Another cytokine mainly secreted by the epithelium is IL‐25 aka IL‐17E, an IL‐17 family cytokine. IL‐25 is overexpressed in bronchial mucosa and the dermis after epithelial exposure to relevant allergens and is up‐regulated in nasal polyps from patients with CRSwNP.^52^, ^53^ Notably, increased IL‐25 production in nasal polyps is associated with corticosteroid sensitivity.^54^, ^55^ Considering asthmatic patients, high expression of IL‐25 evaluated in bronchial biopsies is associated with higher eosinophil infiltration, sputum eosinophils and blood eosinophils.^56^

IL‐33, a member of the IL‐1 cytokine family, is also implicated in type 2 inflammatory responses. It is produced not only by epithelial cells but also by bronchial smooth muscle cells.^57^ IL‐33 strongly elicits the activation of innate type 2 airway immunity, which leads to eosinophil infiltration.^58^ In a mouse model of asthma using an evaluation of bronchial contraction ex vivo, IL‐33 stimulation was correlated with airway hyperresponsiveness.^59^ Increased production of IL‐33 by keratinocytes leads to the expansion of various innate cells in mouse models of AD.^60^, ^61^ Genetic studies in CRSwNP also highlighted the association between IL‐33 and nasal polyposis.^62^

The activation of the immune cascade depends not only on epithelial stimuli but also on professional antigen‐presenting cells such as dendritic cells (DCs). In asthma, airway dendritic cells are essential for inducing naïve T cell commitment towards T helper 2 polarization and subsequent proliferation.^63^ In allergic mouse models of asthma, dendritic cells are critical for inducing robust T helper 2 immunity against house dust mites.^64^ Comparable observations can be made with skin dendritic cells (Langerhans cells), which act as environmental sensors and drive type 2 responses in AD skin.^65^

Type 2 adaptative immunity involves CD4^+^ T helper cells secreting Il‐4, IL‐5 and IL‐13. In asthma, these cytokines can discriminate 2 types of asthma inflammatory profiles: type 2 (or eosinophilic) and non‐type 2.^66^. It has been demonstrated that IL‐13^+^ skin homing Th2 cells were higher in AD patients, particularly those with high IgE phenotypes.^67^ In AD, IL‐4 and IL‐13 lead to skin inflammation, itch and skin hyperpermeability by down‐regulating filaggrin production by keratinocytes.^68^ However, CD4^+^ T helper cells are not the only source of cardinal type 2 cytokines. Group 2 innate lymphoid cells (ILC2s) are also important producers of IL‐5, IL‐9 and IL‐13 depending on the transcription factors GATA‐3 and RORα and the cytokine environment.^69^, ^70^, ^71^, ^72^ Activated ILC2s have been found in greater numbers in the airways of severe eosinophilic asthmatic patients than in mild asthmatic patients and healthy volunteers.^73^ More recently, human studies demonstrated that local allergen stimulation leads to a local infiltration of activated ILC2‐secreting pro‐T2 cytokines.^74^, ^75^ Cooperation between ILC‐2 and T helper 2 cells is sufficient to induce strong type 2 immunity in an adoptive transfer mouse model of asthma.^76^ Moreover, ILC‐2 stimulated by TSLP demonstrates corticosteroid resistance, allowing the sustained secretion of type 2 cytokines despite treatment.^77^ Comparable data are found in eosinophilic nasal polyposis with a higher rate of ILC2s in the mucosa and corticoid sensitivity in these cells.^78^, ^79^ Finally, skin lesions from patients suffering from AD demonstrate a high number of ILC‐2 along with basophils in comparison with healthy skin.^80^

In parallel, in response to IL‐4, specific B cells switch their antibody class towards IgE production. These IgEs are essential to sensitize basophils and mast cells to a specific allergen. In allergy‐driven type 2 immunity, specific IgE is secreted and fixed to high affinity Fc receptors present on mast cells and basophils. Upon a second stimulation, the cross‐linking of fixed IgE leads to the activation of mast cells and basophils and the secretion of presynthesized cytokines. Histamine increases vessel permeability, which leads to tissue oedema. Tryptase, prostaglandins and leukotrienes also increase blood permeability, which favours inflammatory cell migration in tissues. Interestingly, a specific environment can stimulate the production of IgE: in DA, *S*.* aureus* can trigger it by inducing IL‐36 production.^65^ In parallel, specific IgEs also play important role in sensing small amount of allergen, activating and polarizing (type 2 immunity) of the adaptative immune system. Indeed, IgE‐allergen complexes can be internalized by antigen‐presenting cells, like DCs, B cells or even basophils, through IgE high (FcεRI) and low (CD23) affinity receptors and then presented to T cells.^81^, ^82^, ^83^ This facilitated antigen presentation has been implicated in the effector phases allergic rhinitis and allergic asthma and in the immunopathology of atopic dermatitis.^84^, ^85^

Eosinophils are important effectors involved in type 2 immunity. Their production by the bone marrow and their ability to infiltrate tissue are tightly regulated by GM‐CSF, IL‐5, and eotaxin‐1.^86^ In asthma, IL‐5 and eotaxin are critical to eosinophil trafficking to the lung.^87^, ^88^ Along with body mass index and bronchodilator responsiveness, the blood eosinophil count was significantly associated with the exacerbation rate in an exacerbation‐prone severe asthmatic subpopulation.^89^ Conversely, eosinophilic inflammation also correlates with airway obstruction and airway hyperresponsiveness to methacholine.^90^ The production and release of eosinophil basic protein is mainly associated with tissue remodelling and also key features of type 2 disease, such as airway hyperresponsiveness and polyp formation.^91^ However, eosinophilic infiltrates of nasal mucosa and polyps are incompletely associated with symptoms and quality of life.^92^, ^93^ On the other hand, mucosal eosinophils and blood hypereosinophilia are significantly correlated with polyposis recurrence after nasal polypectomy.^94^ In AD, eosinophils are classically elevated in patients' serum and infiltrate lesional skin.^95^ Eosinophils can be detected in cutaneous biopsies from acute and chronic lesions of AD.^96^ However, cutaneous eosinophilia does not seem to correlate with the severity of the disease. Eosinophils seem to be differently associated with severity depending on the involved organ.

Type 2 immunity develops across different environmental barriers following common paths. However, the stimuli and interaction slightly differ from one another. Effector cells and tissue damage are somehow different, and further studies are needed to better understand why.

### 2.3 Targeting type 2 immunity: What have we learned?

Precision medicine has developed in the last 2 decades and aims to personalize therapeutic strategies. Specific biotherapies targeting key pathways of type 2 immunity have been developed and used in the aforementioned diseases, providing interesting insights in real‐life settings (Table 2).

**TABLE 2:** Biotherapies of clinical trials in type 2‐driven airways and cutaneous diseases

Type 2‐related pathway	Targeted therapy	AD	CRSwNP	Asthma	Ref
**IgE**	Omalizumab	No effect	Improved symptoms	Improved symptoms	97, 98, 101, 102
**IL‐5**	Mepolizumab	No effect	Reduced need for surgery	Improved symptoms Reduction of OCS	103, 104, 114, 115
	Benralizumab	*Ongoing* *NCT03563066* *NCT04605094*	*Ongoing* *NCT04157335*	Improved symptoms Reduction of OCS	106, 107, 108
	Reslizumab	No data available	Withdrawn	Improved symptoms	^105^
**IL‐4/Il‐13**	Dupilumab	Improved symptoms	Improved symptoms Reduced need for surgery Reduction of OCS	Improved symptoms Reduction of OCS	116, 117, 118, 119
**IL‐13**	Tralokinumab	Improved symptoms	No data available	No effect	124, 125, 128
	Lebrikizumab	*Ongoing* *NCT04146363* *NCT04178967*	*Ongoing* *NCT04146363* *NCT04178967*	No effect	^123^
**CRTh2**	Fevipiprant	No effect NCT01785602	No data available	No effect	^132^
	AZD1981	No data available	No data available	No effect	^130^
**TSLP**	Tezepelumab	No data available	No data available	Improved symptoms	^136^
**IL‐33**	REGN3500	Ongoing Phase II NCT03736967	Ongoing Combination with dupilumab NCT03112577	No data available	NA

Abbreviations: AD, atopic dermatitis; CRSwNP, chronic rhinosinusitis with nasal polyposis; IgE, Immunoglobulin E, IL, Interleukin; OCS, oral corticosteroids.

The bold text indicates the most common phenotypes of disease and their most common associated comorbidities.

IgE was the first type 2‐related pathway to be targeted with omalizumab, a humanized monoclonal antibody. A phase III study demonstrated that omalizumab significantly improves the asthma exacerbation rate in that population compared with placebo.^97^, ^98^ Routine clinical practice studies have confirmed its positive impact on the control of the disease, quality of life and ability to lower oral corticosteroid therapy.^99^, ^100^ Recent phase III trials of omalizumab in nasal polyposis found significant improvements of symptoms and endoscopic findings under treatment.^101^ Strikingly, even though IgE seemed to be important in descriptive studies, omalizumab failed to show clear efficiency in AD until now, raising the question of its true implication in the disease course.^102^ Whether omalizumab could be efficient in specific AD phenotypes remains to be demonstrated. Indeed, former RCTs used large inclusion criteria without taking into account the different AD endotypes. It cannot be excluded that some AD subpopulations may benefit from omalizumab treatment.

The IL‐5 pathway, mainly associated with eosinophilic involvement, is targeted by the monoclonal antibodies mepolizumab, reslizumab and benralizumab. These drugs are now recommended in severe eosinophilic asthma to reduce the asthma exacerbation rate and oral corticosteroid background treatment.^103^, ^104^, ^105^, ^106^, ^107^, ^108^, ^109^ Such treatments remain efficient in the long term (1 year and beyond), but some data are in favour of a loss of effect after discontinuation, for example, considering mepolizumab.^110^, ^111^, ^112^, ^113^ The IL‐5 pathway seems to be important in symptom development, but sustained untargeted parallel stimuli may explain such recrudescence after discontinuation. In CRSwNP, a phase III study (SYNAPSE) recently demonstrated that mepolizumab significantly improved the total nasal polyp score and nasal obstruction in comparison with placebo.^114^ A randomized clinical trial to assess the efficacy and safety of benralizumab in eosinophilic CRSwNP is ongoing (NCT04157335). In contrast, mepolizumab failed to demonstrate significant clinical effects on AD at 2 weeks in a small RCT.^115^ Benralizumab phase 2 clinical trials are recruiting patients with moderate to severe AD (NCT04605094, NCT03563066). The results from such trials would help define the relevance of targeting eosinophils in AD.

Another approved biologic that can be used to focus on type 2 inflammation is dupilumab. This fully human monoclonal antibody targets IL‐4 receptor α, blocking IL‐4 and IL‐13 signalling. Phase III clinical trials demonstrated a significant reduction in the exacerbation rate and efficient tapering of oral corticosteroid treatment in moderate to severe asthma.^116^, ^117^ Comparable results were observed in CRSwNP, with a significant reduction in nasal symptoms and endoscopic scores and improved quality of life under treatment compared to placebo.^118^ Likewise, in uncontrolled moderate‐to‐severe AD, dupilumab significantly increased the proportion of patients with an Investigator General Assessment (IGA) score of 0 or 1 or a reduction ≥2 under treatment compared to placebo.^119^ Whether targeting the IL‐4/IL‐13 pathway has sustained effects over time and truly changes the disease course remains to be demonstrated. Interestingly, a main side effect of dupilumab is a transient increase in blood eosinophilia independent of the treatment response. Strikingly, data from phase 3 studies demonstrated that a high rate of conjunctivitis could be observed under treatment in AD patients, whereas such side effects seem to be less frequent in patients treated for asthma or CRSwNP.^120^ Few and small series have been published in that subject with the more frequent presentations described are tarsal and bulbar conjunctivitis, blepharitis and limbitis.^121^, ^122^ Small studies suggest that AD severity at baseline and history of conjunctivitis may be risk factors of developing such side effect under dupilumab. Treatment associates artificial tears, topical steroids topical immunosuppressive treatment (tacrolimus ointment, ciclosporine drops). In most severe cases with weak therapeutic response, dupilumab cessation is needed. Until now, to our knowledge, the underlying immune mechanisms remains poorly understood.

Moreover, biologics attempting to directly target IL‐13 (tralokinumab and lebrikizumab) failed to show significant efficiency in asthma.^123^, ^124^, ^125^ Strategies to find suitable biomarkers (blood eosinophils, FeNO, serum periostin) to select potential responders were designed but did not succeed.^126^ In contrast, phase 3 clinical trials recently demonstrated a significant improvement of AD under treatment with tralokinumab with or without topical steroids compared with topical steroids alone or placebo.^127^, ^128^ Promising results from a phase II study considering lebrikizumab in moderate‐to‐severe AD led to the recent onset of 2 parallel phase III clinical trials (ADVOCATE 1 and 2, NCT04146363, NCT04178967).^129^ These different response profiles highlight that despite common pathophysiological pathways, it is still difficult to predict how individual responses occur both between type 2 inflammatory diseases and within a specific disease.

Beyond canonical type 2 cytokines, promising new targets have been discovered. Chemoattractant receptor‐homologous molecule expressed on Th2 cells (CRTh2) is activated by prostaglandin 2, promoting the chemotaxis and activation of Th2 lymphocytes and eosinophils. In a proof‐of‐concept study, AZD1981, an oral CRTh2 antagonist, failed to show a significant increase in peak expiratory flow (primary endpoint), but trends towards an improvement in asthma control score were seen in moderate‐to‐severe asthma patients.^130^ Other data from atopic asthmatic patients display a trend towards an increase in FEV1 under treatment.^131^ In parallel, fevipiprant, another oral CRTh2 antagonist, failed to achieve a significant improvement in severe asthma considering the rate of exacerbation.^132^ Comparable failure was observed considering the targeting of CRTH2 in AD (NCT01785602).^133^ In AD, an innovative strategy has been explored with the nemolizumab, a monoclonal antibody directed against IL‐31 Receptor α subunit, that demonstrated a significant symptomatic improvement of pruritus and cutaneous inflammation compared to placebo plus topical agents.^134^ Indeed, IL‐31, an IL‐6 family cytokine member mainly secreted by Th2 cells, acts not only on peripheral nerve cells (pruritus) but also on immune cells (mast cells, granulocytes) by enhancing their ability to secrete pro‐inflammatory cytokines such as IL‐4 and IL‐13.^135^

Finally, trials assessing the ability of anti‐alarmins to restrict the initiation of type 2 responses are recruiting. In severe asthma, tezepelumab, an anti‐TSLP antibody significantly reduced the annualized rate of asthma exacerbation.^136^ Interestingly, such improvement was observed independently of blood eosinophils (≥ or <300/mm^3^). In parallel, an antibody targeting IL‐33, REGN3500, is currently being tested in AD and asthma as monotherapy or in combination with dupilumab (NCT03736967, NCT03112577, NCT03736967). Moreover, astegolimab, a monoclonal antibody targeting IL‐33 receptor ST2, recently demonstrated in a phase 2b clinical trial a reduction of annualized asthma exacerbation rate in treated severe asthmatics compared to placebo.^137^ Nevertheless, to our knowledge, no anti‐IL‐25 antibodies are currently being tested in clinical trials. The results from biotherapies targeting alarmins are awaited to evaluate the efficiency of stopping epithelial upstream signals to prevent type 2 immunity sustainment.

## 3 TYPE 2 INFLAMMATION: UNCERTAINTIES AND UNMET NEEDS

Although its pathophysiology is now better understood, some aspects of type 2 inflammation remain misunderstood (Table 3). First, some discrepancies exist between observational data and results from clinical trials. For example, IgE seems to be important in AD, but its targeting has no effect in clinical settings.^102^ In parallel, the true pathophysiological role of eosinophils in the aforementioned diseases can be discussed in anti‐IL5 trials.^138^, ^139^, ^140^ Why decreasing eosinophils in type 2 respiratory diseases is efficient in reducing symptoms but not in cutaneous counterparts is an interesting question to be answered. Conversely, targeting both IL‐4 and IL‐13 is efficient in improving symptoms in AD and CRSwNP.^141^, ^142^ Nevertheless, conflicting results between asthma and AD while aiming specifically at IL‐13 may raise the question of potential IL‐4‐driven and IL‐13‐driven diseases.

**TABLE 3:** Unmet needs in type 2‐driven diseases

Category	Unmet need	Needed research	Expected outcome
Pathophysiology	Identification of determinants of the nuances of type 2 inflammation	Direct comparison of pathophysiology between type 2‐driven diseases	Identification of new targets. Identification of critical disease‐modifying traits
Diagnosis	Clear and consensual tools and biomarkers to assess type 2 inflammation	Large‐scale clinical and omics studies	Clear and consensual guidelines for type 2 inflammation assessment
Treatment	Clear biomarker to guide biotherapy choice Global assessment of response of type 2 diseases to biotherapies	Predicting real‐life clinical studies involving multiple biotherapies Multiple type 2 disease studies involving multiple biotherapies	Multiparametric score predicting response to biotherapy Phenotype‐guided treatment Global care of patients suffering from type 2 diseases

Another issue to be settled is the relevance of assessing type 2 inflammation involvement with biomarkers. A biomarker is a molecule, a gene or a characteristic that is linked to a specific diagnosis, prognosis or response to treatment. To be useful, a biomarker must be not only relevant and consistent but also specific and easy to use. Dealing with type 2 inflammation assessment in clinical practice, 2 situations must be emphasized. First, a clinician should be able to recognize type 2 involvement in individual settings. Second, tools should be available to help the clinician choose the best specific treatment for the patient.

In asthma, type 2 extended evaluation is principally done in rare severe forms of the disease (4% of the adult asthma population) due to the complex procedure involved and the cost of the biotherapies.^143^, ^144^ However, such consideration must be tempered, as research on an allergic component has long been part of asthma healthcare strategies (avoidance of allergens, AIT). Phenotyping and endotyping of asthma of any severity is an important objective to improve our knowledge of the natural course of the disease and also its treatment. Consensual eosinophilic severe asthma definition was proposed by a European Respiratory Society taskforce based on a combination of major and minor criteria.^145^ This definition is based mainly on 3 biomarkers: sputum induction, blood eosinophilia and F_ENO_ (exhaled nitric fraction).^146^ Sputum induction is considered to be the gold standard of inflammation evaluation in asthma.^143^ However, this technique requires trained operators and pathologists, lacks standardization and is also restricted to specialized expert centres. Recent guidelines from the European Respiratory Society and American Thoracic Society recommend assessing blood eosinophilia and F_ENO_ at the expense of sputum induction.^109^ Nevertheless, it should be bear in mind that the correlations among blood eosinophilia, F_ENO_, eosinophilic infiltration and symptoms are far from perfect.^147^, ^148^ Conversely, with such obstacles to ensure the strong endotyping of patients, robust biomarkers for the prediction of the response to biotherapies are still lacking. Even though correlations between blood eosinophilia and the response to anti‐IL5 strategies have been separately reported, there are still unexpected non‐responders.^149^, ^150^, ^151^

Considering chronic rhinosinusitis, the main characteristic associated with eosinophilic inflammation is the presence of nasal polyps explored by nasal endoscopy. However, with new efficient therapies available against type 2 immunity pathways, biomarkers and clear strategies are needed. The European Position Paper on Rhinosinusitis and Nasal Polyps 2020 (EPOS 2020) recently positions treatable trait checking, including type 2 inflammation, at the core of secondary and tertiary care of chronic rhinosinusitis.^152^ The use of biotherapy, namely, dupilumab, the only biological currently validated, is restricted to patients with bilateral polyposis who already had endoscopic sinus surgery with a combination of type 2 inflammation evidence, significant symptoms, an inadequate response to medical therapies and/or asthma comorbidity. Along with non‐invasive biomarkers, such as blood eosinophils (≥0.25 G/L) and total IgE (≥100 UI/ml), direct sampling from former surgery allows the direct evaluation of eosinophil involvement in an individual patient (significant if ≥10/high power field) at the cost of relative invasiveness, similar to sputum induction in asthma. Studies are needed to assess the potential of biotherapies to avoid sinus surgery, and robust biomarkers will be needed.

In AD, the use of biomarkers, especially IgE level assessments, is not recommended in routine practice.^12^ Assessing eosinophilic infiltration in skin does not seem to be relevant, as anti‐IL5 biological strategies failed to show sufficient efficacy in AD, questioning the real implication of eosinophils in the disease. Moreover, the efficacy of dupilumab in moderate‐to‐severe extrinsic and intrinsic AD does not argue in favour of type 2 inflammation involvement assessment. However, in clinical research settings, analysis of serum biomarkers allowed the description of four different clusters relative to different inflammatory subtypes (IL‐1R1, Th1/Th2/Th17, Th2/Th22, low Th2/eosinophils).^153^ Conversely, it has been demonstrated that proteomic analysis of tape strips of lesional and non‐lesional skin could draw immune profiles modified by treatment with dupilumab.^154^ Whether further pretreatment exploration of immunity in AD would lead to better healthcare remains to be demonstrated.^155^ Promising results were obtained for fezakinumab, a monoclonal antibody directed against IL‐22, a member of IL‐10 family cytokine whose receptor is mainly expressed on epithelial cells and implicated in proliferation and tissue repair. Indeed, it demonstrated stronger clinical effects and better transcriptomic improvement in severe AD patients with high IL‐22 expression than in those with low IL‐22 expression.^156^ Moreover, interesting results have been obtained using janus kinase (JAK) inhibitors (upadacitinib and baracitinib) in AD by targeting JAK1/2‐STAT6 signalling pathways.^157^

As we presented, there are clear associations between type 2‐driven diseases. They can be successive in time or even concomitant, especially considering chronic rhinosinusitis and asthma. Assuming that some pathophysiological mechanisms are shared, the question of whether a unique treatment targeting type 2 immunity could lead to an overall improvement is interesting. Data already exist with concomitant improvement of asthma and chronic rhinosinusitis. Sinus surgery in patients suffering from chronic rhinosinusitis associated with asthma interestingly improves asthma‐related quality of life.^158^ Asthma control is negatively correlated with the rate of acute exacerbation and recurrence of chronic rhinosinusitis with nasal polyps.^159^, ^160^

Another interesting unsolved question is the potential of treating AD during infancy using type 2‐specific treatment to lower the incidence of respiratory type 2 diseases in adulthood. Due to the incomplete association between those 2 events and the complexity of long‐term longitudinal interventional studies, questions may remain unanswered for a long time. Until now, there is no data supporting a long‐term disease‐modifying effect of biologics in type 2‐driven diseases. A potential explanation of such constatation may be the targeting of specific path without an action in the overall immune response. Such overall sustained action on immune responses has been demonstrated considering allergic immunotherapy (AIT) in allergic asthma and AR.^161^ Besides, first data to be further develop seem to be in favour of preventive effect of AIT in the development of new sensitization and diseases.^162^

Personalized healthcare strategies and biological tools are now shared between respiratory and cutaneous type 2‐driven diseases. Improvements in the evaluation of pathophysiological mechanisms and prediction of treatment responses are needed. Conversely, the opportunity to treat several diseases by targeting common traits is an appealing objective to achieve in the future. However, we still lack evidence to support such strategies. Multidisciplinary evaluation of patients to improve the correct recognition and care of the different type 2 mediated diseases is a first step to achieve such objective. Randomized clinical trials and also pragmatic trials assessing multidisciplinary approach in comparison to standard care are needed.

## 4 CONCLUSION

Respiratory and cutaneous diseases involving type 2 immunity share common clinical and pathophysiological characteristics. While some are concomitant, such as CRSwNP and asthma, consistent with the concept of unified airways, the association between AD and respiratory diseases seems to principally differ. In parallel, the development of new biotherapies targeting specific paths related to type 2 immunity raised new questions. Indeed, discrepancies in clinical responses between the different biotherapies were observed considering the targeting of eosinophils (anti‐IL5) and IgE, raising the question of their true implication in the specific pathophysiology of respiratory and cutaneous diseases. In contrast, the broad positive effect of dupilumab in type 2‐driven pathologies makes us wonder whether some hierarchy in the development of immune dysregulation exists. Studies targeting epithelium upstream signals are ongoing, and their results may contribute to answering this question.

Our conception of the global healthcare of these conditions changed with the rise of personalized and precision medicine in our routine practice. The specific evaluation of relevant targets is now mandatory, but robust biomarkers are still needed. The demonstration of type 2 immunity implications in several other diseases, such as food allergies, eosinophilic esophagitis, chronic urticaria, and atopic keratoconjunctivitis, is now leading to the extension of trials of type 2‐targeting biotherapies. Whether a multidisciplinary approach could possibly improve the overall care of patients suffering from multiple type 2 diseases is a critical question. However, specific interventional studies are needed to obtain strong evidence in favour of this hypothesis.

## CONFLICT OF INTERESTS

DH declares the following conflict of interest: Support for attending meetings and/or travel from SANOFI, Novartis, GSK and AstraZeneca. OM declares the following link of interest: Adviser, and/or clinical study investigator for Medtronic, Sanofi‐Genzyme, GSK, Novartis, MSD, ALK. SB declares the following conflict of interest: Scientific adviser, consultant, and/or clinical study investigator for Almirall, Sanofi‐Genzyme, Abbvie, Novartis, Janssen, Leo‐Pharma, Pfizer, Eli Lilly, UCB Pharma. AM declares the following conflict of interest: Consultant or investigator for Novartis, GSK, SANOFI & Astra Zeneca. LC declares the following link of interest: Support for a PhD.

## AUTHOR CONTRIBUTIONS

DH and LC drafted the manuscript. DH made the figures and tables. OM, SB, AM and LC critically revised the manuscript.

## Data Availability

Data sharing is not applicable to this article as no datasets were generated or analysed during the current study.
